# Characterization of Two Malaria Parasite Organelle Translation Elongation Factor G Proteins: The Likely Targets of the Anti-Malarial Fusidic Acid

**DOI:** 10.1371/journal.pone.0020633

**Published:** 2011-06-10

**Authors:** Russell A. Johnson, Geoffrey I. McFadden, Christopher D. Goodman

**Affiliations:** Plant Cell Biology Research Centre-School of Botany, University of Melbourne, Parkville, Victoria, Australia; Institut National de la Santé et de la Recherche Médicale Cochin, France

## Abstract

Malaria parasites harbour two organelles with bacteria-like metabolic processes that are the targets of many anti-bacterial drugs. One such drug is fusidic acid, which inhibits the translation component elongation factor G. The response of *P. falciparum* to fusidic acid was characterised using extended SYBR-Green based drug trials. This revealed that fusidic acid kills in vitro cultured *P. falciparum* parasites by immediately blocking parasite development. Two bacterial-type protein translation elongation factor G genes are identified as likely targets of fusidic acid. Sequence analysis suggests that these proteins function in the mitochondria and apicoplast and both should be sensitive to fusidic acid. Microscopic examination of protein-reporter fusions confirm the prediction that one elongation factor G is a component of parasite mitochondria whereas the second is a component of the relict plastid or apicoplast. The presence of two putative targets for a single inhibitory compound emphasizes the potential of elongation factor G as a drug target in malaria.

## Introduction

Numerous anti-bacterials are also effective anti-malarials and are a part of current malaria management programs. For instance, doxycycline is widely prescribed for malaria chemoprophylaxis [1] and clindamycin and doxycycline are recommended for use in ACT therapy in the case of initial treatment failure [2]. Anti-bacterials typically work by blocking vital prokaryotic processes such as DNA replication, RNA transcription, protein translation, or peptidoglycan wall synthesis. The key to the success of anti-bacterials is that they specifically target the fundamental differences between these processes in bacteria and the equivalent processes in humans and other eukaryotes. How anti-bacterials kill eukaryotic malaria parasites is not well understood. Two malaria parasite organelles derived from endosymbiotic bacteria, the mitochondrion and the relict plastid (or apicoplast), are the likely targets of these compounds.

Mitochondria occur in most eukaryotes and derive from an alpha proteobacterium that entered into an endosymbiotic partnership with a host cell at the outset of eukaryotic evolution [3]. Though much reduced, mitochondria retain clear hallmarks of their bacterial ancestry, and their housekeeping and metabolic activities are essentially bacterial in nature. Plastids are typically the site of photosynthesis in algae and plants and derive ultimately from endosymbiotic cyanobacteria. A vestigial plastid that lacks the ability to photosynthesize was identified more than a decade ago in malaria parasites [4] and is indispensable for parasite survival [5]. It is now clear that non-photosynthetic plastids occur in almost all members of the Phylum Apicomplexa, where they are referred to as apicoplasts. Recently, a photosynthetic apicoplast was discovered in an apicomplexan symbiont of corals [6], confirming the hypothesis that the ancestors of apicomplexan parasites were photosynthetic autotrophs and converted to parasitism of animals early on in their evolutionary radiation. Apicoplasts are reminiscent of mitochondria in that they retain numerous hallmarks of their bacterial ancestry but their metabolic suite is markedly different, being derived from a different lineage of bacterial ancestors-the photosynthetic cyanobacteria.

Fusidic acid is a potent, narrow spectrum steroid anti-bacterial derived from the fungus *Fusidium coccineum* [7]. It is often used in conjunction with rifampicin to treat severe Gram-positive bacterial infections, such as methicillin-resistant *Staphylococcus aureus* (MRSA). Fusidic acid targets elongation factor G (EF-G), a GTPase critical to the translocation step of bacterial protein synthesis [8]. Fusidic acid binds to EF-G on the ribosome and prevents the EF-G:GDP complex from leaving the ribosome, effectively stalling protein synthesis by steric inhibition [9]. Fusidic acid exhibits anti-*Plasmodium* activity *in vitro* [10], but it has never been utilized as an anti-malarial and nothing is known about its mode of action.

Both the apicoplast and the mitochondrion apparently maintain bacterial-style translation machineries that are candidate targets for fusidic acid. Hard evidence for translation has only been obtained for the *Plasmodium falciparum* apicoplast courtesy of an antibody directed against elongation factor Tu that is encoded on the apicoplast genome [11]. No such evidence for translation in malaria parasite mitochondria exists, but assays of their predicted enzymatic activity suggest that the three proteins encoded by the *Plasmodium* mitochondrial genome are indeed manufactured within the mitochondria [12]. This evidence is supported by the presence of ribosomal RNAs [13] and a host of nucleus-encoded bacterial-like translation components that are targeted to the mitochondrion [14].

With a view to further defining the likely target of fusidic acid in malaria parasites, we explore whether either or both of these organellar translation systems utilize EF-G. Drug trials confirmed the anti-parasitic activity of fusidic acid but demonstrate different characteristics to other bacterial translation inhibitors. Searches of the *P. falciparum* genome for EF-G encoding genes identified two candidates. Bioinformatic analysis and protein sequence comparisons of the predicted protein sequence indicate that one candidate gene is of mitochondrial origin and the other is of a plastid origin. The two gene products localize to the predicted organelles, further confirming that protein translation is occurring in both the apicoplast and mitochondria and suggesting that one or both of these proteins is the target of fusidic acid.

## Results

### Fusidic acid kills *P. falciparum* in the first cycle

**Figure 1:**
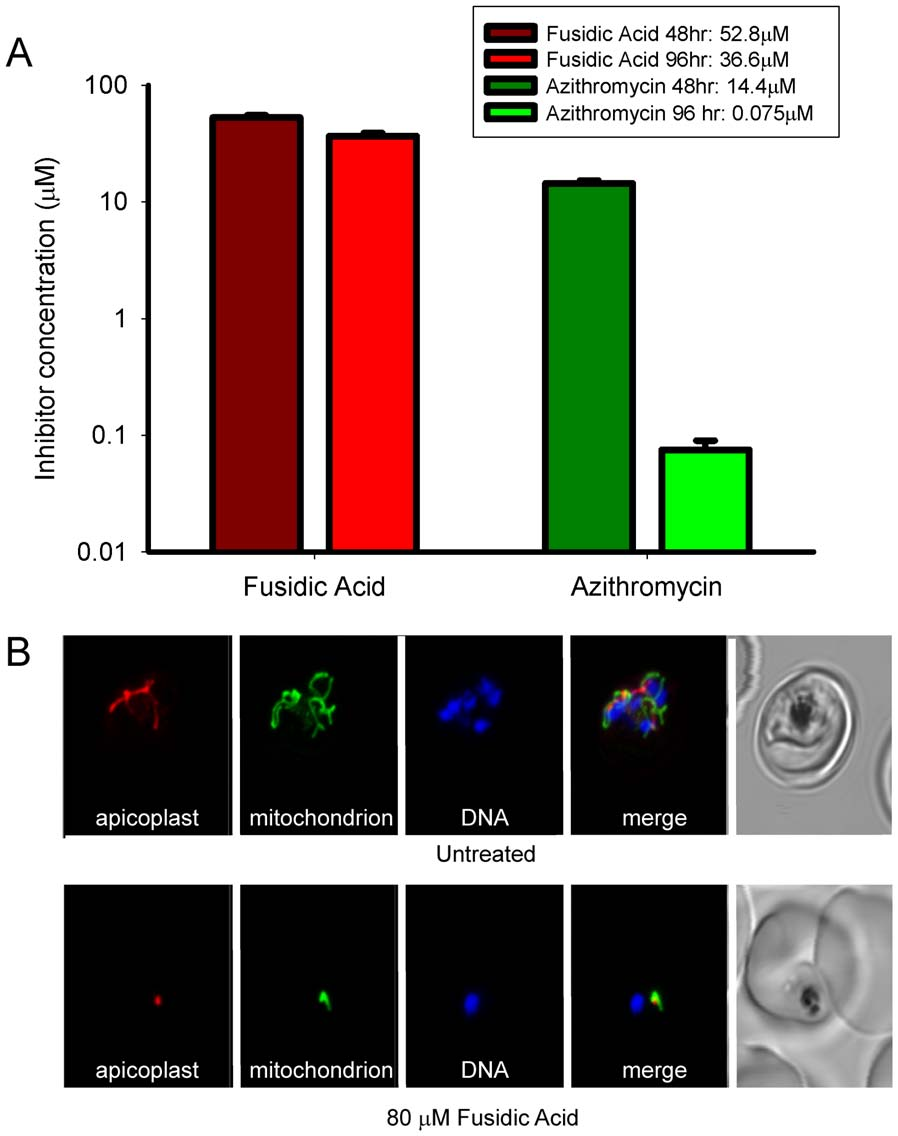
Fusidic acid kills malaria parasites within the first life cycle. A. Parasiticidal activity of fusidic acid on *in vitro* cultured erythrocyte stages of *Plasmodium falciparum*. Fusidic acid kills parasites with an IC_50_ of 52.8 µM (S.E. 2.5, n = 3) after 48 hours (one life cycle). After drug treatment over two erythrocytic cycles, the IC_50_ of fusidic acid (36.6 µM S.E. 2.4, n = 3) is not significantly lower as compared the difference seen for azithromycin (14.4 µM (+/−0.85) v. 0.0750 µM (+/−.015), n = 3); death of malaria parasites is immediate and not delayed. B. Synchronised ring-stage parasites expressing RFP targeted to the apicoplast (red) and YFP targeted to the mitochondria (green) were exposed to i) 80 µM of fusidic acid (IC_90_) or left untreated ii) for 40 hours, the nuclei were stained with Hoescht 33342 (blue) and viewed by confocal laser microscopy.

Most drugs targeting translation in the apicoplast produce a delayed effect in which the treated parasites grow and replicate normally for one life cycle following drug treatment, then die after invading a new blood cell [15], [16], [17]. To test for this drug response we determined the IC_50_ for fusidic acid in *in vitro* cultures of the *P. falciparum* line D10. The IC_50_ values after one life cycle (∼48 hours) are similar to those previously reported [10] and only slightly higher than the values found after two cycles (∼96 hours) (Fig. 1A) indicating that fusidic acid's effects are immediate. This contrasts with other translation-blocking anti-bacterials, such as azithromycin, clindamycin and tetracycline, which exhibit delayed death and have dramatically lower IC_50_ values at 96 hours compared to 48 hours (Fig. 1A, [15], [17]).

Examination of parasite lines exposed to fusidic acid at concentrations equivalent to the 96 hour IC_90_ confirmed the immediate effect. Parasites treated with fusidic acid in early ring stages failed to progress beyond the early trophozoite stage. Consistent with this growth arrest is the lack of significant genome replication and a failure of the organelles to elongate (Fig. 1B). This contrasts with exposure to “delayed-death” antibiotics, where treatments with drug concentrations well in excess of the IC_90_ show no effect on parasite or organellar development during the first 48 hours of drug treatment [15], [17]. It was interesting to note that many of the fusidic acid treated parasites remained within the red blood cell after 48 hours of treatment but were no longer detectable by confocal microscopic examination after a further 48 hours and no nuclear division or merozoite formation was observed during this period. The progressive loss of drug-killed parasites may account for the slight reduction in IC_50_ values between 48 and 96 hours, as the assay used does not differentiate between live parasites and those dead and dying parasites remaining within the red blood cell.

### *P. falciparum* encodes two EF-G proteins of bacterial ancestry

Searches of the *P. falciparum* genome identified two candidate EF-G encoding genes (PFL1590c and PFF0115c) both of which contain GTPase domains. PFL1590c encodes a protein of 803 amino acids that, excluding apicomplexan orthologues, is most similar to the EF-G encoding (*fusA*) gene of the delta proteobacterium *Myxococcus xanthus* (Genbank accession NC_008095.1) with which it shares 42 % amino acid identity. PFF0115c encodes a protein of 937 amino acids that, excluding apicomplexan orthologues, is most similar to the EF-G encoding (*fusA*) gene of the actinobacterium *Nocardioides sp*. (Genbank accession NC_008699.1), with which it shares 46 % amino acid identity. The presence of two bacterium-like EF-G proteins in *P. falciparum* is suggestive of an organellar localisation for these proteins and is consistent with the parasites sensitivity to fusidic acid.

### Alignments of putative *P. falciparum* nucleus-encoded EF-G proteins reveals that the two proteins are distinct

We aligned the two *P. falciparum* EF-G protein sequences with characterised and predicted EF-G sequences from other apicomplexans, algae, higher plants, insects and mammals. We also included the sequence of the previously crystallised *Thermus thermophilus* EF-G [18] as a reference for structural features. Accession numbers for protein sequences used in the alignment are listed in Table S1.

**Figure 2:**
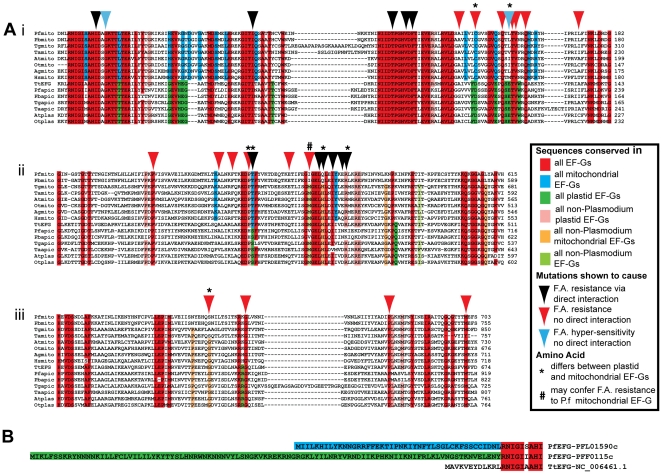
Bioinformatic analysis of *P. falciparum* EF-Gs. A. Sequence alignment of *Plasmodium falciparum* EF-G proteins, selected eukaryotic EF-Gs and the previously crystallised EF-G from the bacterium *Thermus thermophilus.* Only the three regions known to carry fusidic acid resistance mutations are shown. Red boxes indicate amino acids conserved across all EF-Gs. Blue and dark green boxes highlight amino acids conserved within the mitochondrial or plastid EF-Gs, respectively. Light green, orange and pink boxes indicate amino acids conserved in all but the *Plasmodium* EF-Gs, *Plasmodium* mitochondrial EF-Gs and *Plasmodium* apicoplast EF-Gs, respectively. Arrows indicate amino acids known to be involved in fusidic acid activity. Black arrows indicate amino acids that directly interact with fusidic acid. Red and blue arrows indicate amino acids that do not interact directly with fusidic acid but whose mutation can cause resistance and hypersensitivity, respectively. Stars indicate residues that differ between mitochondrial and plastid EF-Gs. The pound sign indicates the only residue in *P. falciparum* mitochondrial EF-G in which the wild type amino acid may confer resistance to fusidic acid. Assignment of EF-Gs to cellular location is based on published studies for *A.thaliana* [20], [22], *H. sapiens* [41] and *O. sativa* [21], [23] and bioinformatic analysis of targeting sequences for the other proteins. B. Sequence alignment of the N-terminal region of the EF-Gs from *P. falciparum* and *T. thermophilus*. Red boxes indicate areas of homology between the three proteins. Blue and green boxes highlight the N-terminal extensions of the two *P. falciparum* proteins used to predict protein targeting.

The alignment of the GTPase domains of these proteins defines two distinct groups, with a number of amino acids conserved within each group but differing between the two (Fig. 2Ai, blue and green boxes). The smaller of the putative *P. falciparum* EF-Gs (PFL1590c) and other predicted mitochondrial EF-Gs clearly group with eukaryotes that do not contain a plastid (i.e. human and *Anopheles* mosquito) and with the EF-G localised to the mitochondria in higher plants [19], [20]. The larger of the two *Pf*EF-Gs (PFF0115c) groups with the plastid-localised EF-G from higher plants [21], [22] and the predicted plastid EF-Gs from other organisms (Fig. 2Ai). The grouping of the mitochondrial and plastid EF-Gs is also evident in alignments of other portions of the protein (Fig. 2A ii, iii), although the overall amino acid conservation in these regions is much lower making the associations less obvious. Indeed, in several instances, specific amino acids are conserved in all EF-Gs except the *Plasmodium* proteins. The grouping of EF-Gs in our alignment correlates with a recently published phylogenetic analysis of bacterial and organellar EF-Gs, which concluded that the phylogenetic signal for the apicomplexan EF-Gs is too weak to draw detailed conclusions about their relationships to other EF-Gs beyond the organellar origin. [23]

To gain insight into the possible targets of fusidic acid in *P. falciparum*, we compared the conservation of amino acids known to confer fusidic acid resistance in various bacteria. These amino acids occur in three areas of the protein corresponding to Figure 2A i, ii and iii [24]. Amino acid residues identified as interacting with fusidic acid in crystallographic studies [25] are largely conserved in all EF-Gs examined, particularly in the GTPase domain (Fig. 2A i, ii black arrows). While several of the fusidic acid interacting amino acids in *P. falciparum* differ from the bacterial residues in the second region, none of the changes correspond to those conferring resistance. They do suggest differences between plastid-localised and mitochondrial EF-Gs (T437-starred) and unique amino acids in the *Plasmodium* EF-Gs (H458, R465-starred).

Other residues that have been correlated with resistance or hypersensitivity but do not interact directly with fusidic acid are highlighted with red and blue arrows, respectively. There is less conservation among these residues than those directly interacting with fusidic acid, but the pattern of conservation in these amino acids is similar to the fusidic acid interacting residues. There are several differences highlighting the separation of mitochondrial and plastid EF-Gs (D109 and E119, starred) and of the *Plasmodium* EF-Gs and all others (P436, M450, G617 – starred). Only one difference suggests enhanced resistance; the amino acid methionine 453 (black pound sign) is altered in the putative *P. falciparum* mitochondrial EF-G to an isoleucine residue that confers limited (4-fold) resistance in *Staphylococcus aureus* [26].

### Bioinformatic analyses suggest that PFL1590c is a mitochondrial-targeted protein and that PFF0115c is an apicoplast-targeted protein

Nucleus encoded organelle-targeted proteins typically contain an N-terminal targeting peptide that directs them either to the *P. falciparum* mitochondrion [27] or apicoplast [28]. Alignment of the two putative EF-G proteins encoded by the *P. falciparum* genes PFL1590c and PFF0155c with EF-G from the bacterium *T. thermophilus* indicated that both malaria parasite proteins bear N-terminal extensions (Fig. 2B). The 44 amino acid N-terminal extension (Fig. 2B green box) of the PFL1590c protein was designated by the *Plasmodium* mitochondrial-targeting prediction program PlasMit [27] to be a likely mitochondrial targeting peptide (Table S2). The 103 amino acid N-terminal extension of PFF0115c (Fig. 2B blue box) is predicted to contain both a signal peptide and a transit peptide by the *Plasmodium* apicoplast targeting prediction program PlasmoAP [29], so PFF0115c is likely an apicoplast-targeted protein (Table S2).

### PFL1590c is a nucleus-encoded mitochondrial-targeted EF-G protein involved in *P. falciparum* mitochondrial translation

**Figure 3 pone-0020633-g003:**
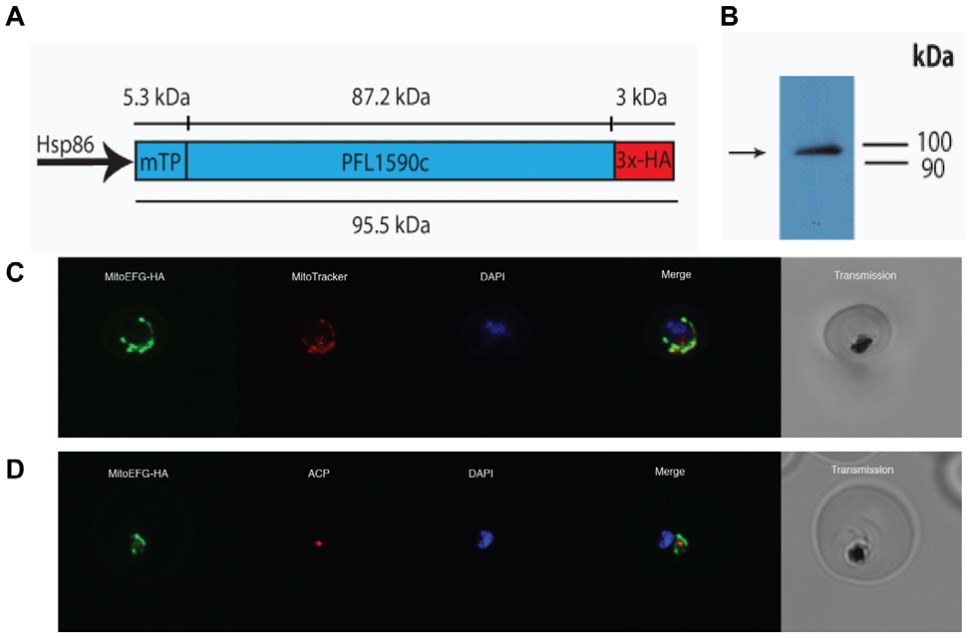
*P. falciparum* EF-G PFL1590c localises to the mitochondrion. A. PFL1590c was fused to a 3x-HA tag to create the construct MitoEFG-HA. B. Western blot analysis of D10 parasites expressing MitoEFG-HA reveals a single band at ∼96 kDa, approximately the mass of the PFL1590c protein fused to a 3x-HA tag. C. An immunofluorescence assay on D10 parasites expressing MitoEFG-HA reveals that PFL1590c is localised to the *P. falciparum* mitochondrion. MitoEFG-HA (green) co-localises with MitoTracker labelling of the mitochondrion (red). DNA stained with Hoescht 33342 (blue). D. An immunofluorescence assay on the MitoEFG-HA expressing parasites counterstained with the ACP antibody as an apicoplast marker [29]. MitoEFG-HA protein localises to the typically elongated mitochondrion (green), but does not co-localise with the punctate apicoplast (red). DNA stained with Hoescht 33342 (blue).

To provide insight into the localisation of PFL1590c, we transfected D10 parasite lines with constructs containing this putative EF-G fused to a 3x hemagglutinin (HA) tag at the C-terminus and driven by the 5′ region of *Pf*HSP86, which we call MitoEFG-HA (Fig. 3A). Western blot analysis of the transgenic parasites confirmed expression of the recombinant protein. A single band of ∼96 kDa was detected, which matches the size of PFL1590c protein with its putative transit peptide and a 3x-HA tag (Fig. 3B). Transit peptides are usually removed after trafficking to the mitochondria and the slight mass anomaly may be due to small differences in predicted and observed molecular mass that are not unusual in polyacrylamide gel electrophoresis.

Immunofluorescence analysis of parasites expressing this episome revealed that the MitoEFG-HA fusion protein is localised to the *P. falciparum* mitochondrion (Fig. 3C). MitoTracker labelling of the mitochondrion co-localised with staining of the MitoEFG-HA protein. Both MitoTracker labelling and antibody staining of the MitoEFG-HA protein reveal a large, crescent shaped organelle characteristic of the *P. falciparum* mitochondrion during the late trophozoite stage of the parasite's asexual cycle [22].

To confirm that the organelle containing PFL1590c is the mitochondrion, we compared the localisation of the MitoEFG-HA fusion protein with the apicoplast resident acyl carrier protein (ACP) [28]. The signal from the HA tagged protein clearly localises to an intercellular compartment distinct from the apicoplast (Fig. 3D), further confirming that PFL1590c is a nucleus-encoded EF-G protein targeted exclusively to the *P. falciparum* mitochondrion.

### PFF0115c is a nucleus-encoded apicoplast-targeted EF-G protein involved in *P. falciparum* apicoplast translation

**Figure 4:**
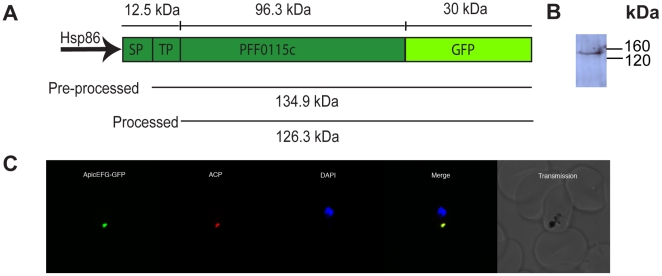
*P. falciparum* EF-G PFF0115c localises to the apicoplast. A. PFF0115c was fused to a C-terminal GFP tag to create the construct ApicEFG-GFP. B. Western blot analysis of D10 parasites expressing ApicEFG-GFP reveals a single band at approximately the predicted molecular mass of the PFF0115c protein attached to a GFP tag. C. An immunofluorescence assay on D10 parasites expressing ApicEFG-GFP reveals that PFF0115c is localised to the *P. falciparum* apicoplast. ApicEFG-GFP (green) co-localises with ACP labelling of the apicoplast (red), and shows the dot-like organelle typical of the *P. falciparum* apicoplast during the ring stage of the asexual cycle [23]. DNA stained with Hoescht 33342 (blue).

Bioinformatic analysis revealed that PFF0115c has all the characteristics of a nucleus-encoded apicoplast-targeted protein. To localise PFF0115c, we transfected constructs containing this putative EF-G fused to a C-terminal GFP in a construct driven by the 5′ region of *Pf*HSP86, which we called ApicEFG-GFP (Fig. 4A). Western blot analysis of parasites expressing ApicEFG-GFP showed a band of between 120 and 160 kDa in mass. This corresponds reasonably well with the predicted molecular mass of the apicoplast EF-G protein fused to GFP (126.3 kDa). Some highly expressed apicoplast-targeted proteins show a second, larger product that corresponds to a preprocessed form of the protein that retains the apicoplast transit peptide while transiting to the apicoplast [30]. No such product was noted on the Western Blot (Fig. 4B). This is not unusual and likely results from the low levels of recombinant protein expression in this experiment.

Immunofluorescence assays on parasites expressing the ApicEFG-GFP episome in conjunction with ACP as an apicoplast marker [28] reveal that PFF0115c is indeed localised to the *P. falciparum* apicoplast (Fig. 4C). Both ACP antibody labelling and ApicEFG-GFP fluorescence revealed a dot-like organelle in the parasite, characteristic of the apicoplast in ring stage parasites during the asexual cycle [31]. These data confirm that the gene annotated as PFF0115c is a nucleus-encoded EF-G protein targeted to the *P. falciparum* apicoplast.

## Discussion

We have shown that fusidic acid, an anti-bacterial that stalls bacterial protein translation by binding to EF-G, kills malaria parasites with an IC_50_ of 52.8 µM. This inhibitory concentration falls within the range seen for other bacterial translation inhibitors when applied for only a single plasmodium life cycle [15], [17], although it is much higher than that seen after prolonged exposure of those compounds causing the delayed-death effect [15], [16], [17]. It is also too high for fusidic acid itself to be an effective drug. However, fusidic acid has only an immediate effect, suggesting that this compound kills *Plasmodium* via a different mechanism that the delayed-death anti-bacterials and may present an effective lead compound for drug development. The inhibitory concentration of any compound reflects many factors, including differences in their ability to come in contact with the target molecule, affinity for the site of action and the ability of the inhibitor to block target activity. All of these factors may be contributing to the IC_50_ of fusidic acid against *P. falciparum* and need to be optimised during further drug development. Determining the specific target of fusidic acid is an important first step in assessing these factors in a systematic way.

Two candidate EF-G proteins that may be fusidic acid targets were identified and localised (Fig. 2–4). Bioinformatic analysis is consistent with these proteins having been introduced as endosymbiont-derived genes that are now located in the parasite nucleus. One EF-G is localised in the parasite mitochondrion, and the second is localised to the relict plastid or apicoplast (Fig. 3,4). Comparisons between primary protein structure of the apicoplast and mitochondrial EF-Gs and sensitive bacterial EF-G proteins suggests that the apicoplast localised EF-G is sensitive to fusidic acid while the mitochondrial EF-G carries a single amino acid residue that confers a weak resistance phenotype in *S. aureus* (Fig. 2,[26]). Although this finding implies that the *P. falciparum* mitochondrial EF-G may be resistant to fusidic acid, differences between the bacterial and *Plasmodium* mitochondrial EF-Gs at other conserved positions makes it difficult to draw specific conclusions about the sensitivity of the *P. falciparum* mitochondrial EF-G to fusidic acid from this single amino acid change without further investigation. A further possibility is that fusidic acid acts by targeting a mechanism unrelated to organellar protein synthesis, but the two EF-Gs identified here represent the most likely targets and require further investigation.

The identification of two nucleus-encoded, organelle localised EF-Gs that could be the target of the same inhibitor presents unique opportunities for the investigation of the effects of organelle specific drugs and in the development of novel anti-malarials. For almost all anti-bacterial compounds currently in use, the target (or predicted target) is encoded on the genome of at least one of the organelles [15], [17], [32] making them refractory to genetic manipulation and difficult to investigate. The presence of nucleus-encoded EF-Gs in both the mitochondrion and apicoplast provides the opportunity to use known bacterial resistance mutations to dissect the effects of specifically blocking protein synthesis in each organelle. It also presents the possibility of developing novel compounds that target EF-G in both organelles, thereby yielding a single anti-malarial compound that could have all the advantages of a multi-drug therapy in terms of avoiding drug resistance.

## Materials and Methods

### Fusidic acids drug trials

Drug trials on *P. falciparum* line D10, were performed as previously described [17]. Parasitemia was assessed using a modified version [17] of the SYBR-Green assay [33]. Inhibitory concentrations were calculated using the Hill-Slop method available in SigmaPlot (Systat software).

### Bioinformatic analysis of EF-G in *P. falciparum*

Protein sequences of the putative mitochondrial EF-G, PFL1590c, and the putative apicoplast EF-G, PFF0115c, were obtained from the *Plasmodium* Genome Resource (www.plasmodb.org) as were sequences for putative homologs from *Plasmodium berghei*. Sequences for the putative *Toxoplasma gondii* EF-G proteins were retrieved using BLAST searches [34] of the *Toxoplasma* Genome Resource (www.toxodb.org). All other sequences were retrieved by text or BLAST searches of GenBank (www.ncbi.nlm.nih.gov) . Alignments were performed and visualised using ClustalX [35]. Accession numbers of EF-G sequences used are included in Table S1. Analysis of N-terminal targeting information was carried out using the *Plasmodium* targeting prediction programs Plasmit [27] and PlasmoAP [29].

### Cloning of PFL1590c and PFF115c localisation constructs

Constructs were created using the MultiSite Gateway system™ (Invitrogen), as previously described [31]. The complete predicted open reading frames of PFL1590c and PFF0115c gene were amplified from *P. falciparum* genomic DNA using the primers shown in Table S3, with attB1 and attB2 sites underlined for the sense and antisense primer, respectively. The PCR products were recombined into the vector pDONR221 according to the manufacturer's instructions (Invitrogen) and fully sequenced. These vectors were then recombined via LR reaction (Invitrogen) to create a C-terminal GFP-tagged PFF0115c construct driven by the *Pf*HSP86 5′ UTR (which we called ApicEFG-GFP) and a C-terminal 3x hemagglutinin (HA)-tagged PFL1590c construct driven by the *Pf*HSP86 5′ UTR (which we called MitoEFG-HA). Both constructs carried a human DHFR expression cassette conferring resistance the drug WR99210 [36].

### Parasite Lines and Transfection

Parasites were grown as previously described [37]. The D10 parasite line was used for all transfections. D10 parasites expressing apicoplast- targeted RFP and mitochondrial-targeted YFP [31] were used for microscopic analysis of drug treatment effects. Transfection of MitoEFG-HA and ApicEFG-GFP plasmids into *P. falciparum* was performed as previously described [38], [39]. Cultures were split 2∶1 14 and 28 days post transfection, yielding drug resistant parasites within 20–30 days.

### Western Blotting

Western blots were performed as previously described [40]. Mouse anti-HA primary antibody was diluted 1/500, and anti-mouse HRP secondary antibody was diluted 1/5000. Rabbit anti-GFP primary antibody was diluted 1/500, and anti-rabbit HRP secondary antibody was diluted 1/5000.

### Immunofluorescence assays

Immunofluorescence assays were performed as previously described [39]. For Mitotracker labelling of the *P. falciparum* mitochondrion, cells were incubated in 20 nM MitoTracker Red (Molecular Probes) diluted in 0/100 RPMI-HEPES medium for 15 mins at 37°C. Cells were washed in 0/100 medium, prior to fixation and labelling. Rat anti-HA primary antibody (Roche) was diluted 1/200. Anti-rat antibody conjugated to Alexafluor 488 (Molecular Probes) was diluted 1/1000. Rabbit anti-ACP antibody [30] was diluted 1/1000, as was anti-rabbit antibody conjugated to Alexafluor 546 (Molecular Probes).

## Supporting Information

Table S1 **Primers used for amplication of PFL1590c and PFF0115c from** ***P. falciparum*** **gDNA.**
(DOC)

Table S2 **Targeting predictions for leaders of PFL0159c and PFF0115c.**
(DOC)

Table S3 **Accession numbers of protein sequences used in alignments.**
(DOC)
