# Supplementary material for: Characterization of Two Malaria Parasite Organelle Translation Elongation Factor G Proteins: The Likely Targets of the Anti-Malarial Fusidic Acid
Source: PLoS One. 2011 Jun 10;6(6):e20633. doi: 10.1371/journal.pone.0020633 (PMC3112199; doi:10.1371/journal.pone.0020633)
Supplement: Table S1 — Primers used for amplication of PFL1590c and PFF0115c from P. falciparum gDNA. (DOC) [file pone.0020633.s001.doc]

## Table S1 - Primers used for amplication of PFL1590c and PFF0115c from *P. falciparum* gDNA.

|  | PFL1590c | PFF0115c |
| --- | --- | --- |
| Sense (attB1) | GGGGACAAGTTTGTACAAAAAAGCAGGCTTACACTTATAATGATTATTT | GGGGACAAGTTTGTACAAAAAAGCAGGCTTAAAATAGAAATGATAAAGCTA |
| Antisense (attB2) | GGGGACCACTTTGTACAAGAAAGCTGGGTATGCTGTACTTTTTTGCTTTAA | GGGGACCACTTTGTACAAGAAAGCTGGGTATTCTTTCTTTTGTAATATTTG |
