# Supplementary material for: Characterization of Two Malaria Parasite Organelle Translation Elongation Factor G Proteins: The Likely Targets of the Anti-Malarial Fusidic Acid
Source: PLoS One. 2011 Jun 10;6(6):e20633. doi: 10.1371/journal.pone.0020633 (PMC3112199; doi:10.1371/journal.pone.0020633)
Supplement: Table S2 — Targeting predictions for leaders of PFL0159c and PFF0115c. (DOC) [file pone.0020633.s002.doc]

**Table** S2 - Targeting predictions for leaders of PFL0159c and PFF0115c

| **Leader** | **Predictor** | **Result** |
| --- | --- | --- |
| First 44 AA of PFL1590c | Plasmit | Jury – 91% yes  Strict - no |
| First 103 AA of PFF0115c | PlasmoAP | Signal Peptide 4/4  Transit Peptide 5/5 |
