# Supplementary material for: Characterization of Two Malaria Parasite Organelle Translation Elongation Factor G Proteins: The Likely Targets of the Anti-Malarial Fusidic Acid
Source: PLoS One. 2011 Jun 10;6(6):e20633. doi: 10.1371/journal.pone.0020633 (PMC3112199; doi:10.1371/journal.pone.0020633)
Supplement: Table S3 — Accession numbers of protein sequences used in alignments. (DOC) [file pone.0020633.s003.doc]

**Table S3 -** Accession numbers of protein sequences used in alignments

| **Abbreviation** | **Organism** | **Accession #** |
| --- | --- | --- |
| Pfmito | *Plasmodium falciparum* 3D7 | XP_001350724 |
| Pbmito | *Plasmodium berghei* strain ANKA | XP_678154 |
| Tgmito | *Toxoplasma gondii* ME49 | XP_002365190 |
| Tamito | *Theileria annulata* strain Ankara | XP_955491 |
| Atmito | *Arabidopsis thaliana* | NP_175135 |
| Otmito | *Ostreococcus tauri* | CAL54657 |
| Agmito | *Anopheles gambiae* Strain PEST | XP_318822 |
| Hsmito | *Homo sapiens* | NP_079272 |
| TtEFG | *Thermus thermophilus* HB8 | YP_144961 |
| Pfapic | *Plasmodium falciparum* 3D7 | XP_966014 |
| Pbapic | *Plasmodium berghei* strain ANKA | XP_680354 |
| Tgapic | *Toxoplasma gondii* ME49 | XP_002366083 |
| Taapic | *Theileria annulata* strain Ankara | XP_954449 |
| Atplas | *Arabidopsis thaliana* | NP_001031452 |
| Otplas | *Ostreococcus tauri* | CAL54715 |
